# Investigating Exposure to Violence and Mental Health in a Diverse Urban Community Sample: Data from the South East London Community Health (SELCoH) Survey

**DOI:** 10.1371/journal.pone.0093660

**Published:** 2014-04-01

**Authors:** Giouliana Kadra, Kimberlie Dean, Matthew Hotopf, Stephani L. Hatch

**Affiliations:** 1 Biomedical Research Centre Nucleus, Department of Psychological Medicine, Institute of Psychiatry, King's College London, London, United Kingdom; 2 School of Psychiatry, University of New South Wales and Justice Health & Forensic Mental Health Network, Sydney, Australia; 3 Department of Psychological Medicine, Institute of Psychiatry, King's College London, London, United Kingdom; University of Stellenbosch, South Africa

## Abstract

**Background:**

General population surveys have seldom examined violence as a multidimensional concept and in relation to an array of mental disorders.

**Methods:**

Data from the South East London Community Health Study was used to examine the prevalence, overlap and distribution of proximal witnessed, victimised and perpetrated violence and their association with current mental disorders. We further investigated the cumulative effect of lifetime exposure to violence on current mental disorders. Unadjusted and adjusted (for confounders and violence) models were examined.

**Results:**

In the last twelve months, 7.4% reported witnessing violence, 6.3% victimisation and 3.2% perpetration of violence. There was a significant overlap across violence types, with some shared correlates across the groups such as being younger and male. Witnessing violence in the past year was associated with current common mental disorders (CMD) and post-traumatic stress disorder (PTSD) symptoms. Proximal perpetration was associated with current CMD, PTSD symptoms and past 12 months drug use; whereas proximal victimisation was associated with lifetime and past 12 months drug use. Lifetime exposure to two or more types of violence was associated with increased risk for all mental health outcomes, suggesting a cumulative effect.

**Conclusion:**

Exposure to violence needs to be examined in a multi-faceted manner: i) as discrete distal and proximal events, which may have distinct patterns of association with mental health and ii) as a concept with different but overlapping dimensions, thus also accounting for possible cumulative effects.

## Background

Violence is a multi-dimensional phenomenon that can be experienced as a victim, witness and perpetrator. Examining its prevalence and associations is necessary to understand violence occurrence [1]. Research on clinical populations has advanced our knowledge by investigating associations between violence and mental disorders. Victimisation in clinical populations has been associated with personality disorder [2] and post-traumatic stress disorder (PTSD) [3]; whereas perpetration has been associated with substance abuse [4] and severe mental illness [5]. However, less is known about the associations between violence and mental disorders in the general population, a gap in the literature that has been recognised by several scholars [6]–[11]. There is emerging evidence demonstrating that exposure to violence (ETV) in urban community settings is associated with mood, anxiety and substance use disorders [8], [58]. More specifically, perpetration has been associated with alcohol and substance misuse [6], [12], [13], whereas witnessed violence and victimisation have been associated with mental illnesses [14], [15] such as depression, anxiety [16] and PTSD [17]. The literature suggests that women exposed to violence generally report more disorders such as depression [18]–[20], whereas men report more externalising behaviours such as alcohol use problems [21]. In the UK, despite significant advances in estimating the annual prevalence of violence (victimisation estimated at 3.1% [22]; perpetration at 5.4% [23] and witnessing at 22% [24]), the relationship with mental disorders in the community is poorly understood.

Existing general population research has been hampered by several limitations. Studies have seldom examined multiple dimensions of violence in the same sample, despite evidence indicating a co-occurrence across victimisation and perpetration [6], [10], [25], and witnessing violence and victimisation [26]. This has limited our ability to draw comparison across ETV types and understand their co-occurrence. Furthermore, there has been a paucity of research examining violence subtypes in relation to both internalising and externalising mental health outcomes simultaneously [27], [58]. In adult and adolescent general populations [8], [27], [57], experiencing more than one dimension of violence has been associated with the severity of recent mental health episodes, suggesting a cumulative effect. A further limitation has been posed by research predominantly focusing on proximal ETV (occurring in the last 12 months) and seldom examining distal (lifetime) ETV, which has also been associated with current mental disorders such as PTSD, depression and substance use [27], even after adjusting for proximal adversities [15], [28]. Therefore, to unpick the association between violence and mental health, it is essential to examine violence in a multi-faceted manner (where proximal and distal subtypes of violence are simultaneously considered in the same general population sample) in relation to an array of mental health outcomes.

In the present study, we examine the prevalence, inter-relationships and associations of different ETV types in a diverse urban population sample: the South East London Community Health (SELCoH) study. On a local level, our sample was similar to the 2011 UK Census data for the boroughs we examined with regards to demographic and socioeconomic indicators. On a national level, the study catchment area has a higher level of deprivation and a level of violence significantly above England's average [29]–[31].

The aims of this analysis were as follows: 1) to estimate the prevalence of proximal witnessing, victimisation and perpetration; and examine their overlap and distribution by socio-demographic and socioeconomic characteristics; 2) to examine the unadjusted and adjusted associations between proximal types of violence and current mental disorders; 3) to examine the cumulative effect of lifetime exposure to violence on current mental health. We hypothesised that witnessed violence and victimisation would be more prevalent than perpetration. We further expected to find an overlap between the different categories of violence and therefore some shared correlates across violence. We anticipated that all categories of violence would have an association with common mental disorders (CMD) and that there would be an interaction with gender, such that women exposed to violence would show a higher prevalence for CMD. We expected to find that lifetime ETV would be associated with current mental disorders, with the association increasing when more than one type has been experienced.

## Methods

### Sample and Procedures

The South East London Community Health (SELCoH) study is an urban, population cross-sectional survey, which aimed to examine psychiatric and physical morbidity in the London boroughs of Southwark and Lambeth. Data were collected between 2008 and 2010. Private households were selected from the Small User Postcode Address File (PAF), using a stratified random sampling approach. Households that were non-residential, shared, vacant or receiving more than 50 item of mail per day were excluded. All adult residents aged 16 year and over, were invited to participate in the survey. A total of 1698 adults from 1075 households were recruited, achieving a within household participation rate of 71.9% and household participation rate of 51.9%. Trained interviewers conducted face to face interviews using a computer assisted interview schedule. All participants were compensated for their time with 15GBP. For detailed information on SELCoH study methods, refer to Hatch *et al*. [32], [33].

### Ethics statement

The SELCoH study received full ethical approval from the King's College London research ethics committee for non-clinical research populations (reference CREC/07/08-152). Written consent was obtained from all participants in the study.

### Measures

The three types of violence were defined by reports of one or more events within each of the following categories: 1) witnessed violence was determined by asking participants if they had: seen something violent happen to someone (e.g. attacked or beaten) or seen someone killed within the last 12 months; 2) victimisation was reported as having experienced within the last 12 months being attacked, mugged, robbed, or been the victim of a serious crime; injured you with a weapon – gun, knife, stick, etc. and hit you, bit you, slapped you, kicked you, or forced you to have sex against your wishes; 3) perpetrated violence was indicated by participants reporting whether or not they had in the last 12 months: attacked or robbed someone; injured someone with a weapon – gun, knife, stick etc. and hit, bit, slapped, or kicked another person [34]. Participants who answered ‘yes’ to one or more question were coded as 1, and all other participants were coded as 0. Therefore, three distinct but potentially overlapping variables were created. We examined lifetime ETV by asking the above questions in relation to incidents which have occurred prior to the last 12 months, thus creating discrete categories of lifetime ETV (no ETV; exposure to one; two; or three types of violence).

### Outcomes

Six measures of participants' current mental health were included. The presence of CMD was established through a structured interview (the Revised Clinical Interview Schedule (CIS-R) [35]), which assesses symptoms for CMD within the last month in 14 domains: fatigue, sleep problems, irritability, worry, depression, depressive ideas, anxiety, obsessions, subjective memory and concentration, somatic symptoms, compulsions, phobias, physical health worries and panic. Each domain contains 4 questions (depressive ideas is an exception and contains five questions) and each question contributes by one point to the total domain score (ranging from 0 to 4). For the purpose of this study, we used the overall score, which is composed by the total scores of all 14 domains. The cut-off score of 12 was used to indicate the presence of CMD [35]. Personality dysfunction (PD) was measured using the Standardised Assessment of Personality- Abbreviated Scale (SAPAS) [36] with specificity of 0.85 and sensitivity of 0.94 in a clinical sample. This measure consists of eight questions on personality traits, which are scored dichotomously as either present or absent. The maximum score is 8 and participants scoring 4 and above were considered to screen positive for PD. We chose a cut off score which is higher than the one used in the clinical population (cut off score of 3), this more conservative approach has been supported by previous work on SAPAS, under the assumption that the prevalence of screening positive for PD in the general population would be lower [37]. PTSD was assessed by the Primary Care PTSD Screen (PC-PTSD) [38], which assesses symptoms present over the last month. The tool consists of 4 items and was developed by the National Centre for PTSD. A cut off score of 3 was chosen based on its good specificity (0.88) and sensitivity (0.76) [38]. The Alcohol Use Disorders Identification Test (AUDIT) [39] was utilised to measure hazardous alcohol use within the last year. The tool was developed by the World Health Organisation (WHO) and generates a score ranging from 0 to 40; an AUDIT score of 8 was considered to indicate a hazardous alcohol use. Participants were asked whether they have used any of the following illicit drugs (e.g. cannabis, amphetamines, cocaine/coke, ecstasy, acid/LSD, tranquilisers, crack and heroin) in their lifetime and in the last 12 months.

### Socio-demographic and socioeconomic factors

A number of socio-demographic and socioeconomic factors were included to describe the distribution of ETV. Age was considered in 10 years interval, after examining the distribution of the data: 16 to 24 years; 25 to 34 years; 35 to 44 years; 45 to 54 years; 55 to 64 years and 65 years and over. Ethnicity categories were determined by self-identification into one of the following groups: White British, Black Caribbean, Black African, Black Other, Indian, Pakistani, Bangladeshi, Chinese or Other. The Black Other, Indian, Pakistani, Bangladeshi, Chinese or Other categories were collapsed for analysis due to small numbers within these groups [32]. In view of the high proportion of migrant population in the sample we examined [32], we accounted for migrant status; this was self-reported and categorised as follows: UK born; living in the UK 0 to 4 years; 5 to 10 years and 11 years or more. These categories were based on historical differences in migration periods [32] and to avoid small cell sizes.

The highest level of educational attainment was reported as one of the following four categories: no qualifications; qualifications up to GCSE or ordinary level; qualifications up to advanced level; and degree or above. Employment status was reported as one of the following: full time; part time/casual; student; unemployed; sick/disabled; retired; and looking after kids. To improve distribution, information was re-categorised into the following categories: 1) employed (including full time, part time and casual); 2) students; 3) unemployed and 4) other (temporary sick or permanent sick/disabled, retired or looking after the home with children). Household income (i.e. gross yearly income from all sources before deductions for income and National Insurance) was presented as a categorical variable in the survey questionnaire, therefore participants self-identified with one of the following categories: £0 to £5,475; £5,476 to £12,097; £12,098 to £20,753; £20,754 to £31,494; and £31,495 and over.

### Statistical analysis

STATA 11 [40] was used to conduct all statistical analyses. We used survey commands (svy) for estimates of prevalence and associations where appropriate to generate robust standard errors. All analyses of SELCoH data accounted for clustering by household inherent in the study design and weighted for within household non-response, comparing all eligible household members (i.e., 16 years or older) by gender and age. For further information on weighting in SELCoH refer to Hatch *et al*. [32]. We reported the unweighted frequencies for the exposure and outcome variables. Pearson's Χ^2^ tests with Rao & Scott second-order corrections with 95 percent confidence intervals were applied for categorical outcomes, p values were reported where appropriate. Bivariate associations between potential confounders and the categories of violence were examined using univariate logistic regression. Multivariate logistic regression models were employed to examine the associations between proximal ETV types and the categorical outcomes. Odds ratios (OR) and 95 per cent confidence intervals (CI) were reported for all associations. Models were adjusted as follows: model 1 was adjusted for known confounders of violence and mental health [2], [3], such as age (used as a continuous variable) and gender; model 2 was further adjusted for other possible confounders, after considering factors relevant to our sample, such as ethnicity, employment, education, household income and migrant status; and model 3 was further adjusted for the remaining categories of violence, thus accounting for possible association between the ETV types [26]. We tested for the interaction of gender with violence subtypes in relation to the mental health outcomes, on the basis of previous literature [2], [3], [19], [21] indicating that the effect of violence on mental health differs by gender. Lastly, a multivariate logistic regression was conducted to examine the relationship between distal ETV and current mental disorders. This model excluded all violence that has occurred in the last year. All models were adjusted for socio-demographic and socioeconomic confounders as described above.

## Results

**Figure 1:**
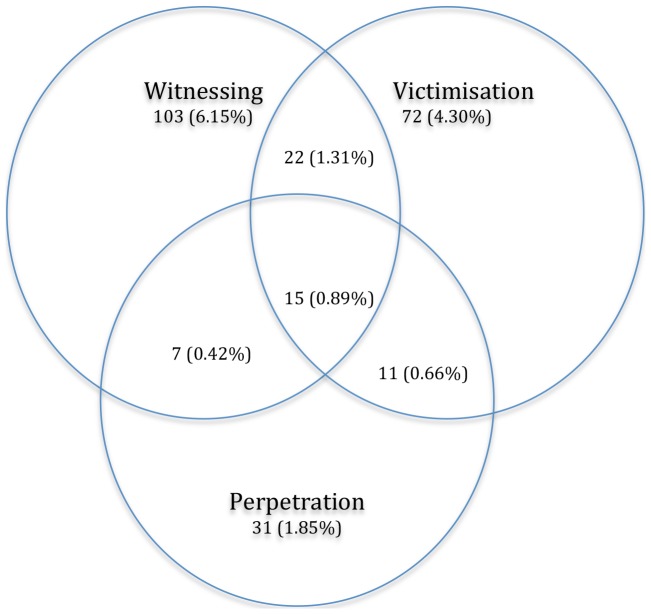
Past year exposure to violence categories. Figure 1 describes the co-occurrence of witnessing, victimisation and perpetration in the last 12 months. There were 1698 participants in the total sample. Out of those, in the last 12 months, 6.15% reported they have exclusively witnessed violence; 4.30% had been victimised; and 1.85% reported solely perpetration. An estimated 0.66% reported victimisation and perpetration of violence; 1.31% reported witnessing violence and victimisation; 0.42% reported witnessing and perpetration and 0.89% reported all three ETV types.

Figure 1 demonstrates the occurrence and overlap of proximal exposure to violence. Table 1 summarises the weighted prevalence of violence categories and their overlap- proximal witnessing of violence was reported by 7.4% of the participants, victimisation by 6.3% and perpetration by 3.2%. The weighted prevalence of exposure to both victimisation and perpetration of violence was 0.5%; 1.2% reported witnessing violence and victimisation; 0.3% reported witnessing and perpetration and 0.7% reported all three ETV types.

**Table 1:** Prevalence estimates for proximal violence^a^ by socio-demographic and socioeconomic characteristics.

Type of exposure to violence		Witnessed		Victimised		Perpetrated	
n		147		120		64	
% (95% CI)		7.4 (6.13–8.68)		6.3 (5.12–7.44)		3.2 (2.42–4.02)	
Characteristics	n (%)	% (95% CI)	p-value	% (95% CI)	p-value	% (95% CI)	p-value
**Exposure to violence**							
Witnessed	147(8.74)	NA		24.9 (17.58–32.35)		13.7 (8.01–19.31)	
Victimised	120 (7.13)	29.4 (20.79–38.05)		NA		19.4 (12.26–.52)	
Perpetrated	64 (3.81)	31.2 (19.72–42.69)		37.9 (25.43–50.32)		NA	
**Age**							
16–24	356 (20.97)	18.9 (14.41–23.33)	<0.001	14.3 (10.73–17.90)	<0.001	10.1 (6.94–13.20)	<0.001
25–34	404 (23.79)	9.6 (6.71–12.48)		6.3 (3.82–8.85)		3.3 (1.50–5.07)	
35–44	336 (19.79)	4.7 (2.36–7.08)		4.5 (2.28–6.66)		1.7 (0.43–2.99)	
45–54	264 (15.55)	4.7 (2.12–7.21)		5.3 (2.54–7.99)		1.7 (0.19–3.29)	
55–64	163 (9.60)	2.3 (−0.27–4.91)		4.6 (1.00–8.26)		1.2 (−0.05–3.01)	
65+	175 (10.31)	1.3 (−0.17–2.76)		1.3 (−0.14–2.73)		-	
**Gender**							
Female	959 (56.48)	6.1 (4.55–7.77)	<0.01	5.4 (3.96–6.87)	<0.03	2.7 (1.73–3.66)	0.07
Male	739 (43.52)	10.0 (7.89–12.11)		8.0 (6.11–9.93)		4.3 (2.83–5.74)	
**Ethnicity**							
White	1,051 (61.97)	6.4 (4.81–7.96)	0.11	6.6 (5.09–8.13)	0.18	3.4 (2.32–4.39)	0.69
Black Caribbean	143 (8.43)	8.1 (3.11–13.11)		9.1 (4.13–13.98)		3.1 (0.37–5.74)	
Black African	234 (13.80)	11.0 (7.24–14.85)		5.4 (2.61–8.28)		3.9 (1.53–6.26)	
Other	268 (15.80)	8.3 (5.24–11.35)		3.9 (1.73–6.16)		2.2 (0.54–3.80)	
**Migrant Status**							
UK born	1,010 (60.77)	7.5 (5.78–9.20)	0.08	7.4 (5.74–8.98)	0.06	3.9 (2.82–5.14)	<0.05
0–4 years	137 (8.24)	9.7 (5.02–14.40)		7.6 (3.44–11.74)		2.2 (0.80–4.39)	
5–10 years	178 (10.71)	11.2 (6.38–16.05)		3.2 (0.65–5.81)		2.3 (0.64–4.44)	
11+ years	337 (20.28)	5.3 (2.95–7.67)		4.3 (2.09–6.61)		1.3 (0.25–2.40)	
**Household Income**							
£0–£5,475	139 (9.68)	9.7 (4.69–14.72)	0.21	7.9 (3.09–12.66)	0.36	3.3 (1.67–5.93)	0.51
£5,476–£12,097	212 (14.76)	9.3 (5.45–13.21)		5.9 (2.92–8.83)		3.9 (1.50–6.39)	
£12,098–£20,753	203 (14.14)	5.7 (2.77–8.55)		5.8 (2.41–9.18)		2.5 (0.06–4.32)	
£20,754–£31,494	179 (12.47)	4.8 (1.79–7.85)		9.0 (4.67–13.41)		3.3 (1.01–5.58)	
£31,495 and over	703 (48.96)	6.5 (4.66–8.32)		5.1 (3.49–6.64)		2.1 (1.02–3.15)	
**Education**							
No qualifications	228 (13.58)	4.7 (2.29–7.15)	<0.01	3.3 (1.04–5.62)	<0.001	3.2 (1.27–5.16)	<0.001
GCSE	332 (19.77)	8.4 (5.51–11.24)		9.7 (6.61–12.81)		4.9 (2.76–7.04)	
A-level's	426 (25.37)	10.8 (7.57–14.06)		9.6 (6.69–12.58)		5.4 (3.22–7.62)	
Degree or above	693 (41.27)	6.1 (4.34–7.91)		4.0 (2.60–5.41)		1.2 (0.39–1.96)	
**Employment**							
Employed	921 (54.53)	5.9 (4.44–7.49)	<0.001	5.4 (3.89–6.87)	<0.001	2.0 (1.11–2.99)	<0.001
Students	247 (14.62)	21.7 (15.91–27.53)		13.8 (9.44–18.12)		10.6 (6.72–14.53)	
Unemployed	170 (10.07)	11.5 (6.52–16.49)		10.2 (5.44–14.89)		5.4 (2.09–8.79)	
Other	351 (20.78)	2.2 (0.70–3.61)		3.3 (1.47–5.05)		1.1 (0.21–1.99)	

a witnessed, victimised and perpetrated categories are overlapping.

As described by Table 2, all categories of violence were strongly associated with younger age. All three categories were more frequent in men, but for perpetration this result was not statistically significant. There was no difference in reporting violence by ethnicity, with the exception of witnessed violence which was most commonly reported by participants who identified with Black African ethnicity. Participants who had migrated to the UK five or more years previously reported lower levels of victimisation and perpetration in comparison to UK born participants. With regards to socioeconomic indicators, there was no difference in reporting violence by household income. However, all types of violence were significantly more prevalent amongst participants reporting education of GCSE or A-Level and unemployed or student work status. Further analyses (not shown) established that, with the exception of the association between witnessed violence and education, age did not account for these associations.

**Table 2:** Bivariate analysis of the association between proximal violence^a^ and socio-demographic and socioeconomic characteristics.

Type of exposure to violence	Witnessed	Victimised	Perpetrated
n	147	120	64
% (95% CI)	7.4 (6.13–8.68)	6.3 (5.12–7.44)	3.2 (2.42–4.02)
Characteristics	Unadjusted OR (95% CI)	Unadjusted OR (95% CI)	Unadjusted OR (95% CI)
**Age**			
16–24	1	1	1
25–34	0.46 (0.29–0.71)**	0.41 (0.24–0.68)***	0.30 (0.16–0.59)***
35–44	0.21 (0.12–0.39)***	0.28 (0.16–0.50)***	0.16 (0.07–0.37)***
45–54	0.21 (0.11–0.39)***	0.33 (0.18–0.61)***	0.16 (0.06–0.41)***
55–64	0.10 (0.03–0.33)***	0.29 (0.12–0.69)**	0.11 (0.02–0.49)**
65+	0.06 (0.02–0.18)***	0.07 (0.02–0.25)***	-
**Gender**			
Female	1	1	1
Male	1.70 (1.19–2.43) **	1.52 (1.04–2.23)*	1.62 (0.96–2.71)
**Ethnicity**			
White	1	1	1
Black Caribbean	1.29 (0.63–2.66)	1.40 (0.74–2.68)	0.91 (0.35–2.37)
Black African	1.82 (1.14–2.91)*	0.81 (0.44–1.49)	1.16 (0.57–2.37)
Other	1.33 (0.82–2.15)	0.58 (0.31–1.09)	0.64 (0.28–1.47)
**Migrant Status**			
Born in the UK	1	1	1
0–4 years	1.33 (0.73–2.40)	1.03 (0.55–1.96)	0.55 (0.19–1.54)
5–10 years	1.56 (0.90–2.69)	0.42 (0.18–0.99)*	0.56 (0.19–1.57)
11years or more	0.69 (0.41–1.17)	0.57 (0.32–1.04)	0.32 (0.14–0.78)*
**Household Income**			
£0–£5,475	1.55 (0.81–2.96)	1.60 (0.77–3.35)	1.59 (0.60–4.25)
£5,476–£12,097	1.48 (0.86–2.56)	1.17 (0.62–2.19)	1.93 (0.84–4.42)
£12,098–£20,753	0.86 (0.46–1.61)	1.15 (0.57–2.32)	1.19 (0.47–3.01)
£20,754–£31,494	0.73 (0.35–1.51)	1.86 (0.99–3.49)	1.59 (0.65–3.91)
£31,495 and over	1	1	1
**Education**			
No qualifications	0.76 (0.41–1.42)	0.82 (0.37–1.83)	2.79 (1.11–6.99)*
GCSE	1.40 (0.86–2.28)	2.57 (1.54–4.29)***	4.32 (1.91–9.78)***
A-level's	1.85 (1.18–2.92)**	2.55 (1.56–4.17)***	4.81 (2.16–10.72)***
Degree or above	1	1	1
**Employment**			
Employed	1	1	1
Students	4.37 (2.82–6.77)***	2.81 (1.76–4.49)***	5.68 (3.03–10.67)***
Unemployed	2.05 (1.17–3.58)*	1.99 (1.11–3.58)*	2.75 (1.25–6.07)*
Other	0.35 (0.16–0.73)**	0.59 (0.31–1.12)	0.53 (0.21–1.37)

a witnessed, victimised and perpetrated categories are overlapping.

*p<0.05,

**p<0.01,

***p<0.001.

### Proximal ETV and current mental health

Table 3 summarises the prevalence estimates of proximal violence and current mental disorders, whereas Table 4 demonstrates the association between proximal violence and mental health. The overall prevalence of CMD was 24.2%. Amongst individuals reporting ETV, CMD was highest in those reporting perpetration. The unadjusted models suggested that all ETV types were associated with increased odds for CMD. However adjusting for potential confounders led to a slight attenuation in all associations; the addition of other categories of violence fully attenuated the associations for victimisation but not for witnessing and perpetration in relation to CMD. The overall proportion of participants who screened positive for PD was 15.3%. Participants reporting victimisation and perpetration had the highest proportion of those who screened positive for PD. Overall, proximal ETV was not associated with PD screen status. Adjusting for age and gender appeared to augment the association between victimisation and screening positive for PD; however this was not maintained following further adjustment for socioeconomic factors. Similarly, adjusting for socioeconomic factors boosted the association between perpetration and screening positive for PD; however this effect was attenuated after adjusting for the remaining types of violence. An estimated 5.5% of all participants screened positive for PTSD, with all categories having a similar prevalence of PTSD symptoms. Proximal exposure to any type of violence was associated with increased odds for PTSD symptoms. After adjusting for the co-occurrence of violence, there was a slight attenuation for witnessing violence and full attenuation for victimisation and perpetration.

**Table 3:** Prevalence estimates for proximal exposure to violence^a^ and current mental health.

Type of Exposure to Violence		No ETV b	Witnessed c	Victimised d	Perpetrated e
n		1416	147	120	64
% (95% CI)		85.2 (83.45–86.95)	7.4 (6.13–8.68)	6.3 (5.12–7.44)	3.2 (2.42–4.02)
Outcome	n	% (95% CI)	% (95% CI)	% (95% CI)	% (95% CI)
Common Mental Disorder	396	22.3 (19.93–24.79)	32.7 (24.58–40.81)	37.6 (28.28–46.95)	44.4 (31.64–57.21)
Personality Dysfunction	241	14.8 (12.82–16.84)	17.7 (11.07–24.37)	22.0 (13.80–30.26)	22.1 (11.27–32.98)
Post Traumatic Stress Disorder	89	4.8 (3.56–5.97)	10.0 (4.21–15.85)	11.4 (5.27–17.47)	11.4 (3.37–19.35)
Lifetime Drug Use	864	45.4 (42.3–48.56)	56.5 (47.41–65.55)	64.5 (55.29–73.74)	63.3 (50.12–75.14)
Drug Use in last 12 months	363	15.5 (13.56–17.52)	32.3 (24.02–40.53)	45.2 (35.92–54.58)	47.8 (33.97–59.58)
Hazardous Alcohol Use	343	15.6 (13.60–17.74)	29.7 (21.59–37.80)	33.8 (25.36–42.31)	37.0 (25.24–48.82)

a witnessed, victimised and perpetrated categories are overlapping.

b this group has not been used as a reference as it includes solely participants who have said ‘no’ to all ETV types.

c the reference group is all participants who have not witnessed violence in the past 12 months.

d the reference group is all participants who have reported no victimisation in the past 12 months.

e the reference group is all participants who have not perpetrated violence in the past 12 months.

**Table 4:** Multivariate logistic regression of the association between proximal exposure to violence^a^ and current mental health.

Type of Exposure to Violence	Witnessed c	Victimised d	Perpetrated e
Outcome	OR (95% CI)	OR (95% CI)	OR (95% CI)
**Common Mental Disorder**			
Unadjusted Model	1.60 (1.09–2.37)*	2.00 (1.32–3.03)***	2.63 (1.55–4.46)***
Model 1^f^	1.68 (1.13–2.51)**	2.10(1.38–3.21)***	2.79 (1.62–4.80)***
Model 2^g^	1.88 (1.19–2.98)**	1.76 (1.08–2.85)*	2.45 (1.25–4.77)**
Model 3^h^	1.63 (1.02–2.60)*	1.47 (0.89–2.43)	2.03 (1.03–3.98)*
**Personality Dysfunction**			
Unadjusted Model	1.21 (0.75–1.96)	1.62 (0.98–2.68)	1.61 (0.85–3.05)
Model 1 f	1.34 (0.82–2.16)	1.75 (1.10–2.90)*	1.78 (0.92–3.41)
Model 2 g	1.23 (0.71–2.13)	1.45 (0.81–2.62)	2.09 (1.03–4.21)*
Model 3 h	1.10 (0.63–1.92)	1.28 (0.68–2.41)	1.89 (0.93–3.87)
**Post Traumatic Stress Disorder**			
Unadjusted Model	2.06 (1.03–4.13)*	2.39 (1.25–4.59)**	2.32 (1.02–5.29)*
Model 1 f	2.17 (1.02–4.63)*	2.47 (1.26–4.84)**	2.37 (1.00–5.62)*
Model 2 g	2.93 (1.31–6.51)**	2.41 (1.09–5.31)*	3.36 (1.24–9.06)*
Model 3 h	2.55 (1.15–5.64)*	1.62 (0.68–3.85)	2.65 (0.90–7.81)
**Lifetime Drug Use**			
Unadjusted Model	1.51 (1.04–2.21)*	2.15 (1.42–3.26)***	1.92 (1.12–3.30)*
Model 1 f	0.95 (0.64–1.41)	1.59 (1.03–2.47)*	1.17 (0.66–2.04)
Model 2 g	1.76 (1.03–2.98)*	2.20 (1.19–4.05)**	2.15 (0.78–5.87)
Model 3 h	1.49 (0.87–2.50)	1.93 (1.02–3.63)*	1.73 (0.61–4.93)
**Drug Use in last 12 months**			
Unadjusted Model	2.29 (1.55–3.40)***	4.19 (2.84–6.19)***	4.19 (2.47–7.10)***
Model 1 f	1.27 (0.84–1.93)	2.96 (1.93–4.54)***	2.32 (1.35–4.00)**
Model 2 g	1.38 (0.87–2.21)	3.12 (1.87–5.20)***	2.88 (1.46–5.67)**
Model 3 h	1.06 (0.62–1.79)	2.78 (1.60–4.83)***	2.19 (1.03–4.62)*
**Hazardous Alcohol Use**			
Unadjusted Model	2.11 (1.41–3.17)***	2.59 (1.74–3.84)***	2.88 (1.72–4.84)***
Model 1 f	1.35 (0.86–2.12)	1.89 (1.23–2.91)**	1.84 (1.15–3.19)*
Model 2 g	1.59 (0.95–2.65)	1.86 (1.03–3.37)*	1.39 (0.66–2.96)
Model 3 h	1.44 (0.85–2.45)	1.72 (0.92–3.21)	1.07 (0.46–2.47)

a witnessed, victimised and perpetrated categories are overlapping.

c the reference group is all participants who have not witnessed violence in the past 12 months.

d the reference group is all participants who have reported no victimisation in the past 12 months.

e the reference group is all participants who have not perpetrated violence in the past 12 months.

f model adjusted for age and gender.

g model adjusted for age, gender, ethnicity, employment, education, household income and migrant status.

h model adjusted for all confounders and for the other two categories of violence.

*p<0.05,

**p<0.01,

***p<0.001.

Lifetime drug use was reported by 46.8% of participants in the sample. Amongst individuals reporting ETV, those who have been victimised in the past year reported the highest proportion of lifetime drug use. Although the unadjusted models indicated that proximal ETV was associated with increased odds for lifetime drug use, the adjusted models indicated that this association was solely maintained for victimisation. Drug use in the past 12 months was reported by 18.1% of all participants, with the highest portion being amongst the perpetrated group. Both unadjusted and adjusted models indicated that proximal exposure to victimisation and perpetration was associated with increased odds for past 12 months drug use. The association with witnessed violence was fully attenuated after controlling for socio-demographic factors. Hazardous alcohol use was reported by 17.5% of all participants, with the highest proportion observed in the perpetrated group. Unadjusted odd ratios suggested an association between all ETV categories and hazardous alcohol use. However, the association with witnessing violence was fully attenuated after adjusting for socio-demographic factors. Similarly, adjusting for socioeconomic factors appeared to fully attenuate the association between perpetration and hazardous alcohol use in model 2. For victimisation, adjusting for violence co-occurrence attenuated the association.

We examined the interaction terms for ETV categories and gender on CMD. Our results suggested no interaction for proximal witnessing (p = 0.34, not shown), victimisation (p = 0.48, not shown), and perpetrations (p = 0.94, not shown). On further examination (Table S1), we also detected no gender interaction with proximal ETV on the remaining mental health outcomes.

### Lifetime ETV and current mental health

**Figure 2:**
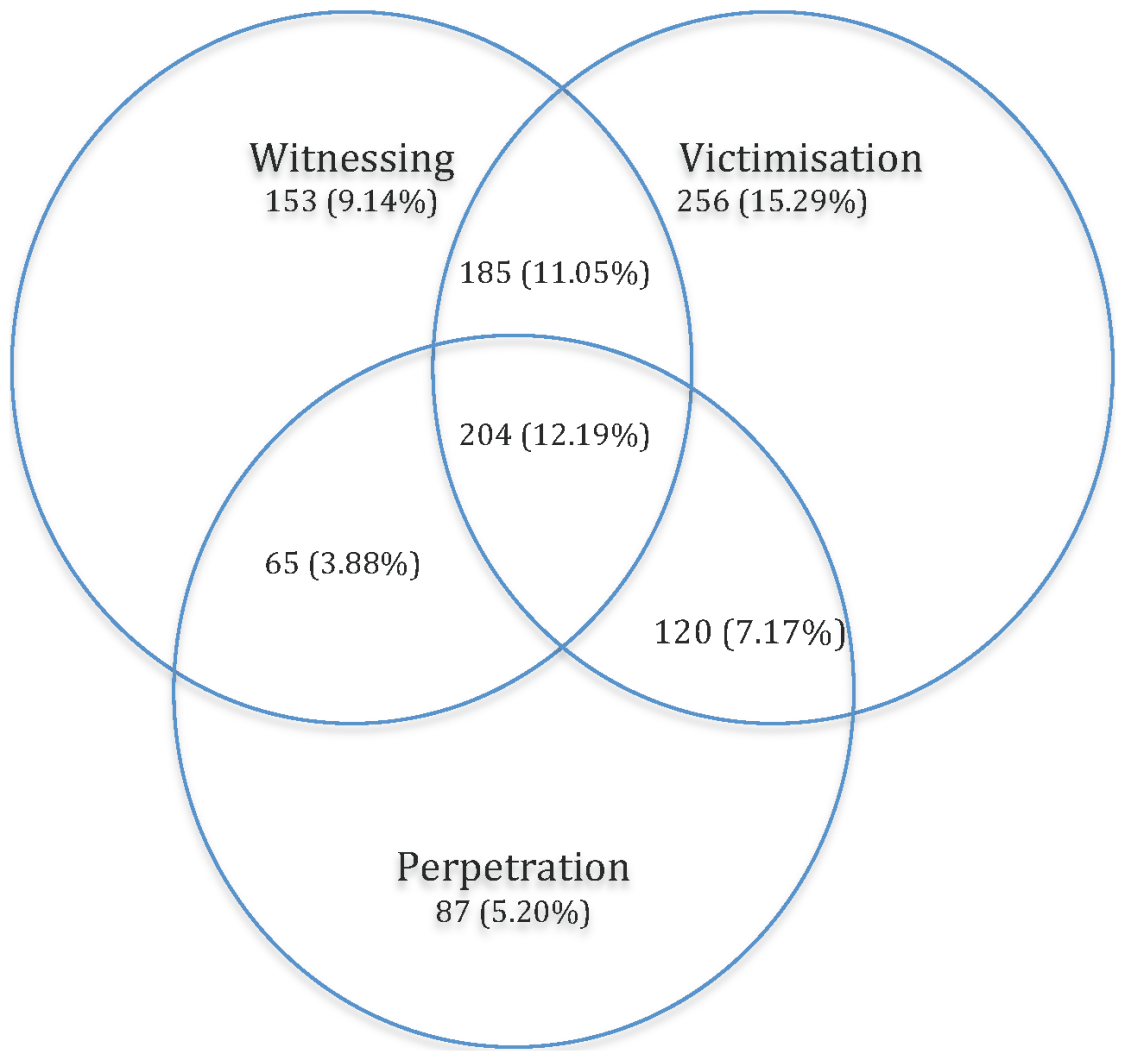
Lifetime exposure to violence categories. Figure 2 describes the co-occurrence of witnessing, victimisation and perpetration previously to the last 12 months. There were 1698 participants in the total sample. Out of those, prior to the last 12 months, 9.14% had solely witnessed violence; 15.29% had been victimised; 5.20% had perpetrated violence. Out of the total sample, 7.17% have experienced victimisation and perpetration; 11.05% reported witnessing violence and victimisation; 3.88% have witnessed and perpetrated violence and 12.19% have experienced all three types of violence.

Figure 2 summarises the occurrence and overlap of lifetime ETV in the sample, whereas Table 5 demonstrates the weighted prevalence of lifetime ETV and current mental health. As demonstrated in Figure 2 and Table 5, there was a notable co-occurrence of lifetime ETV in the sample. The weighted prevalence of participants who have experienced one, two or three types of violence was 30.1%, 21.3% and 10.8% respectively. Of those, 6.6% have experienced victimisation and perpetration; 11.4% reported witnessing violence and victimisation; and 3.2% have witnessed and perpetrated violence. Approximately 37.8% of the sample reported no lifetime ETV.

**Table 5:** Prevalence estimates for lifetime exposure to violence and current mental health.

Number of ETV types		No ETV i	1 type of ETV	2 types of ETV	3 types of ETV
n		604	496	370	204
% (95% CI)		37.8 (35.20–40.41)	30.1 (27.74–32.39)	21.3 (19.24–23.32)	10.8 (9.34–12.35)
Outcome	n	% (95% CI)	% (95% CI)	% (95% CI)	% (95% CI)
Common Mental Disorder	396	15.4 (12.24–18.63)	23.8 (19.83–27.83)	33.4 (28.19–38.65)	33.7 (26.79–40.69)
Personality Dysfunction	241	10.9 (0.82–13.52)	12.9 (9.89–16.05)	21.9 (17.16–26.56)	24.3 (17.72–30.94)
Post Traumatic Stress Disorder	89	1.8 (0.75–2.88)	4.4 (2.50–6.35)	8.9 (5.79–12.10)	14.1 (8.85–19.40)
Lifetime Drug Use	864	31.7 (27.69–35.78)	49.5 (44.59–54.37)	59.7 (54.31–65.18)	68.5 (61.71–75.39)
Drug Use in last 12 months	363	10.3 (7.94–12.71)	17.8 (14.19–21.48)	25.1 (20.64–29.56)	33.9 (27.18–40.71)
Hazardous Alcohol Use	343	12.2 (9.58–14.89)	16.5 (12.96–20.00)	24.2 (19.83–28.56)	26.9 (20.59–33.23)

i No ETV has been used as the reference group.

Table 6 demonstrates the association between exposure to one or more types of lifetime violence and current mental disorders. Lifetime exposure to one, two and three categories of violence was associated with increased odds for CMD, lifetime and past 12 months drug use, with evidence of a gradient across the groups (p<0.001, not shown). Although a similar pattern was observed for PTSD symptoms, the association with exposure to one category of violence was fully attenuated after adjusting the model for socioeconomic factors. Trend analyses of the unadjusted and adjusted model 1 suggested a gradient across the categories (p<0.001, not shown). Lifetime exposure to two or more categories of violence was also associated with increased odds for screening positive for PD and hazardous alcohol use. On further examination, we established that experiencing both victimisation and perpetration; or witnessed violence and victimisation (not shown) was independently associated with a greater risk for screening positive for PD, whereas for hazardous alcohol use experiencing victimisation and perpetration; or witnessing and perpetration was associated with increased risk.

**Table 6:** Multivariate logistic regression of the association between lifetime exposure to violence and current mental health.

Number of ETV types		No ETV i	1 type of ETV	2 types of ETV	3 types of ETV
Outcome	n	OR (95% CI)	OR (95% CI)	OR (95% CI)	OR (95% CI)
**Common Mental Disorder**	396				
Unadjusted Model		1	1.71 (1.23–2.38)***	2.75 (1.97–3.83)***	2.79 (1.89–4.12)***
Model 1 f		1	1.78 (1.28–2.54)***	3.10 (2.29–4.47)***	3.38 (2.25–5.10)***
Model 2 g		1	1.77 (1.21–2.57)**	3.04 (2.08–4.42)***	2.86 (1.82–4.49)***
**Personality Dysfunction**	241				
Unadjusted Model		1	1.22 (0.84–1.79)	2.29 (1.56–3.39)***	2.64 (1.68–4.16)***
Model 1 f		1	1.26 (0.86–1.84)	2.49 (1.68–3.73)***	3.05 (1.91–4.89)***
Model 2 g		1	1.41 (0.90–2.19)	2.93 (1.82–4.70)***	3.81 (2.19–6.61)***
**Post Traumatic Stress Disorder**	89				
Unadjusted Model		1	2.50 (1.18–5.31)*	5.31 (2.67–10.59)***	8.89 (4.23–18.71)***
Model 1 f		1	2.61 (1.22–5.56)*	6.08 (3.01–12.26)***	11.22 (5.18–24.32)***
Model 2 g		1	2.48 (0.96–6.42)	7.27 (3.07–17.15)***	13.38 (5.13–34.90)***
**Lifetime Drug Use**	864				
Unadjusted Model		1	2.11 (1.63–2.73)***	3.19 (2.41–4.23)***	4.69 (3.28–6.71)***
Model 1 f		1	2.19 (1.69–2.87)***	3.31 (2.48–4.41)***	4.47 (3.10–6.53)***
Model 2 g		1	2.36 (1.69–3.29)***	2.75 (1.90–3.97)***	4.13 (2.62–6.48)***
**Drug Use in last 12 months**	363				
Unadjusted Model		1	1.88 (1.33–2.66)***	2.91 (2.08–4.07)***	4.46 (3.04–6.55)***
Model 1 f		1	1.95 (1.37–2.77)***	2.97 (2.09–4.21)***	4.14 (2.74–6.24)***
Model 2 g		1	1.71 (1.15–2.53)**	2.53 (1.71–3.74)***	3.59 (2.22–5.83)***
**Hazardous Alcohol Use**	343				
Unadjusted Model		1	1. 42 (0.99–2.00)	2.29 (1.64–3.19)***	2.64 (1.78–3.92)***
Model 1 f		1	1.38 (0.97–1.97)	2.10 (1.49–2.95)***	2.13 (1.41–3.23)***
Model 2 g		1	1.30 (0.87–1.93)	1.92 (1.31–2.81)***	1.75 (1.10–2.78)*

f model adjusted for age and gender.

g model adjusted for age, gender, ethnicity, employment, education, household income and migrant status.

i No ETV has been used as the reference group.

*p<0.05,

**p<0.01,

***p<0.001.

## Discussion

Our results are from a study, which provided rich data derived from a densely populated and diverse metropolitan area. The findings indicate violence co-occurrence in both proximal and distal exposure and the presence of some shared correlates across types, suggesting that individuals at risk of one type are at increased risk of experiencing other types of violence. Conversely, there were some distinct patterns of association with mental disorders that persisted after adjusting for potential confounders and the remaining categories of violence. This suggests that certain ETV could be associated with particular constellations of mental health symptoms. Our results further indicate that the impact of violence is long-standing, with the effect on current mental health also evident for distal ETV.

In comparison to previous general population surveys [22]–[24], we detected a lower prevalence of witnessing and perpetration and a higher level of victimisation. The latter finding was not entirely unexpected if we consider studies that have examined victimisation in greater detail [14] and a community population of high deprivation [41]. However it was unexpected to find a lower prevalence for witnessing and perpetration. As predicted, we detected an overlap across categories for both distal and proximal violence; witnessing and victimisation emerged as the most frequently overlapping categories [9], [26], with a small proportion of participants also experiencing all types of violence. As previously reported, we found that certain groups such as those who are younger; male; of unemployed and student work status [17], [22], [24], [42], [43] are at an increased risk for all categories of proximal violence. Shared correlates across categories and violence co-occurrence suggest that certain individuals in the general population could be particularly vulnerable to ETV; routinely recording this information could be valuable in identifying individuals who are at risk.

In contrast with previous research (indicating that victimisation is more common amongst individuals who self-identify with non-white ethnicity [22] and perpetration amongst individuals who self-identified with White and Mixed ethnicity [44]) we found no difference in proximal ETV across ethnic groups with the exception of participants who self-identified with Black African ethnicity who were more likely to report witnessing violence in the last 12 months. The SELCoH study sample has the advantage of examining a population with a higher proportion of ethnic minorities and migrant population that has been previously under-researched [32]. In addition, Black African and Black Caribbean ethnicities have seldom been examined separately [22]. These findings suggest that future research may benefit from further investigating ethnicity in similar population samples and in greater detail, particularly with regard to ethnicities previously considered as similar and aggregated in the same group. With regard to migrant status, we found no considerable differences in ETV across the groups, though victimisation and perpetration was less common amongst participants who have lived in the UK for five or more years, in comparison to UK born participants. Although previous research [45], [46] has established a relatively high ETV in migrant population, studies have predominately examined violence prior to migration. Therefore, more studies with substantial migrant populations should consider pre and post migration violence separately.

The relationship between ETV and mental disorders emerged as a complex one. Some distinct patterns of association emerged for proximal witnessing and perpetration with CMD and proximal witnessing with PTSD symptoms [27], [28], [47], [48]; proximal perpetration and past year drug use [13], [49]; and proximal victimisation and lifetime and past 12 months drug use [27]. Conversely, violence co-occurrence had an important effect on all associations by either partially or fully attenuating the observed associations with mental disorders. This interplay between violence and mental disorders is consistent with recent evidence [6], [58], similarly indicating that although violence has an independent association with some externalising mental disorders in the general population; victimisation is one of the factors which explains the relationship between violent behaviour and certain mood disorders. Therefore, examining the individual effect of violence is not sufficient to fully understand its relationship with mental disorders, it is thus imperative to consider co-occurrence of ETV categories.

Our results further indicate that violence requires to be examined in longitudinal fashion, where ETV prior to the last 12 months could have an independent (of proximal violence) and cumulative effect on some current mental disorders. Although previous research has been limited, existing evidence is consistent with our findings [16], [28]. In contrast to proximal ETV, we also found that distal exposure to two or more types of violence is also associated with less common outcomes such as screening positive for PD and hazardous alcohol use. This appears consistent with clinical guidelines where long-standing and pervasive behaviour is integral to reaching a diagnosis of PD [50].

Lastly, we found no gender effect for proximal ETV and CMD. In clinical populations, gender is known to be an important modifying factor in associations between serious mental illness and both violent victimisation and perpetration [3]. General population research has been less consistent [13], [51]. There is some existing evidence demonstrating that women are at an increased risk of reporting sexual victimisation and mental health difficulties [52], [53], whereas men who experience victimisation are more likely to report alcohol misuse [21].

### Limitations

As a result of the cross sectional nature of this study, we are unable to make causal inference with regard to the relationship between ETV and mental disorders. It is possible that some recall bias has occurred in the study as a result of lifetime ETV reports being based on participants' recollection. Despite a satisfactory within household participation rate (71.9%, [32]), it is possible that participants who were most unwell or distressed, or with the most serious history of ETV did not take part in the study, thus introducing non-response bias [54]. This may account for the lower prevalence of proximal witnessed and perpetrated violence than were detected in previous surveys. In addition, we examined a diverse urban population with relatively high crime rates, therefore generalising the findings beyond this population needs to be done with caution.

Future studies may benefit from a more detailed assessment of ETV. Our questions on violence were part of a wider survey on health, and were not detailed - they did not distinguish between different sub-types of violence (i.e. sexual victimisation), the seriousness of violence, its frequency, its context, or the relationship between victim and perpetrator. It is feasible that the above factors have a distinct, nevertheless an important effect on the prevalence of violence and its relationship with mental disorders. For example, repeat exposure to one type of violence has been associated with internalising mental disorders [55] and alcohol use [21]. Therefore, future research should consider the effect of multiple exposures to violence within a single category in more detail, as well as the cumulative effect of exposure to more than one category of violence on mental health. Similarly, the exposure to a particularly serious type of violence has also been associated with detrimental effects on mental health [56]. Therefore, a more detailed assessment of violence would have allowed the categories we assessed to be expressed as dimensions of severity of violence. In contrast with previous research [10], [14] we did not examine less severe forms of violence such as threatened or attempted physical assault. It is likely that this has contributed to detecting a lower prevalence for certain ETV types. With regard to the mental health measures utilised in this study, we did not have an available standardised measure for illicit substance abuse. Therefore, our results do not necessarily reflect a problem with illicit substances. In addition, PTSD symptoms were not measured in relation to the ETV event; therefore we cannot assume a link.

## Conclusion

Our results highlight the need to examine violence in a multidimensional manner accounting for a diverse range of violence experiences and inclusive of perpetration. Future research would benefit from a more detailed assessment of violence, where ETV is also examined as discrete distal and proximal events and as overlapping dimensions, thus accounting for their cumulative effect. Our findings suggest that there is a complex relationship between ETV and mental disorders in the general population, which warrants further investigation. More specifically, examining the needs of individuals experiencing multiple dimensions of violence and mental disorder could be invaluable in informing service development plans and initiatives to improve assessment and intervention for this group.

## Supporting Information

Table S1 Gender interaction with proximal ETV types by mental health outcome.
(DOCX)
