# Supplementary material for: Investigating Exposure to Violence and Mental Health in a Diverse Urban Community Sample: Data from the South East London Community Health (SELCoH) Survey
Source: PLoS One. 2014 Apr 1;9(4):e93660. doi: 10.1371/journal.pone.0093660 (PMC3972242; doi:10.1371/journal.pone.0093660)
Supplement: Table S1 — Gender interaction with proximal ETV types by mental health outcome. (DOCX) [file pone.0093660.s001.docx]

| **ETV type** | **Witnessing** |  | **Victimisation** |  | **Perpetration** |  |
| --- | --- | --- | --- | --- | --- | --- |
| **Mental health outcome** | **OR (95% CI)** | **p-value** | **OR (95% CI)** | **p-value** | **OR (95% CI)** | **p-value** |
| Common Mental Disorder | 0.70 (0.34- 1.45) | 0.34 | 0.75 (0.34- 1.66) | 0.48 | 1.04 (0.35- 3.07) | 0.94 |
| Personality Dysfunction | 1.30 (0.56- 3.02) | 0.54 | 0.58 (0.21- 1.61) | 0.29 | 1.12 (0.30- 4.15) | 0.87 |
| Post Traumatic Stress Disorder | 0.51 (0.14- 1.84) | 0.31 | 0.42 (0.09- 1.81) | 0.24 | 1.19 (0.23- 6.30) | 0.83 |
| Lifetime Drug Use | 1.63 (0.79- 3.37) | 0.18 | 1.08 (0.48- 2.41) | 0.85 | 1.08 (0.37- 3.12) | 0.89 |
| Drug Use in last 12 months | 0.94 (0.41- 2.15) | 0.89 | 0.53 (0.25- 1.16) | 0.11 | 0.64 (0.23- 1.81) | 0.40 |
| Hazardous Alcohol Use | 0.46 (0.21- 1.03) | 0.06 | 0.59 (0.27- 1.31) | 0.19 | 1.13 (0.39- 3.29) | 0.82 |
